# Diphenyl Ditelluride: Redox-Modulating and Antiproliferative Properties

**DOI:** 10.1155/2019/2510936

**Published:** 2019-10-24

**Authors:** Cristiano Trindade, André Luiz Mendes Juchem, Temenouga N. Guecheva, Iuri M. de Oliveira, Priscila dos Santos Silveira, José Eduardo Vargas, Renato Puga, Claudia Ó. Pessoa, João A. P. Henriques

**Affiliations:** ^1^Facultad de Ciencias Básicas y Biomédicas, Universidad Simón Bolívar, Barranquilla, Colombia; ^2^Departamento de Biofísica/Centro de Biotecnologia, Instituto de Biociências, Universidade Federal do Rio Grande do Sul, Porto Alegre, Brazil; ^3^Instituto de Cardiologia do Rio Grande do Sul/Fundação Universitária de Cardiologia, Porto Alegre, Brazil; ^4^Instituto de Ciências Biológicas (ICB)-Universidade de Passo Fundo, Passo Fundo, Brazil; ^5^Clinical Research Center, Hospital Israelita Albert Einstein (HIAE), São Paulo, Brazil; ^6^Departamento de Fisiologia e Farmacologia, Universidade Federal do Ceará, Fortaleza, Brazil; ^7^Laboratório de Cultura de Células, Programa de Pós-graduação em Biotecnologia, Universidade do Vale do Taquari, Lajeado, Brazil

## Abstract

Tellurium is a rare element that has been regarded as a toxic, nonessential element, and its biological role is not clearly established. In addition, the biological effects of elemental tellurium and some of its organic and inorganic derivatives have been studied, leading to a set of interesting and promising applications. Diphenyl ditelluride (DPDT), an organic tellurium derivate, showed antioxidant, antigenotoxic, antimutagenic, and anticancer properties. The antioxidant and prooxidant properties of DPDT are complex and depend on experimental conditions, which may explain the contradictory reports of these properties. In addition, DPDT may exert its effects through different pathways, including distinct ones to those responsible for chemotherapy resistance phenotypes: transcription factors, membrane receptors, adhesion, structural molecules, cell cycle regulatory components, and apoptosis pathways. This review aims to present recent advances in our understanding of the biological effects, therapeutic potential, and safety of DPDT treatment. Moreover, original results demonstrating the cytotoxic effects of DPDT in different mammalian cell lines and systems biology analysis are included, and emerging approaches for possible future applications are inferred.

## 1. Introduction

Tellurium (Te) is a stable and solid element that pertains to chalcogens (group 16 in the periodic table), which is the same group that includes sulfur, selenium, and polonium. Te is classified as a metalloid because of its features between metals and nonmetals [1, 2]. It was discovered by Franz Joseph Müller von Reichenstein in 1782, 35 years before the lighter, closely related metalloid, selenium, was discovered [1]. In contrast to selenium, sulfur, and oxygen, Te does not have physiological functions in mammalian cell biology [3]; however, some publications have reported that Te is present in body fluids [1]. Whereas Te-containing proteins were not identified in human cells, Te in telluromethionine and tellurocysteine was found in proteins in yeast, fungi, and bacteria [4]. In a comprehensive review of the biological activities of Te compounds, it was pointed out that Te could be facing the same discrimination as selenium once did and that the natural biological functions of Te may be revealed in the future [5].

The industrial applications of inorganic Te compounds include production of nanoparticulate semiconductors and metal-oxidizing solutions [6, 7]. Furthermore, the use of organotellurium compounds in insecticides, magnetic disks, catalysts, and stabilizers is tending to increase [6, 8]. Te was also utilized in the composition of thermoelectric materials and quantum dots for diagnostics and treatment [9].

The risk of human environmental exposure to Te is unpredictable due to its elevated usage [10]. The use of Te in the manufacturing of electronic devices and nanomaterials demands safety risk assessment to deal with the electronic material constituents. Moreover, these materials usually can be numerous toxic elements, explaining why research on the environmental and occupational toxicity of these materials has been widely conducted [11–14]. The biological functions of elemental Te have been a matter of interest although few studies examining the toxicity of its ionic forms have been conducted [15, 16]. In the environment, Te can be (bio)methylated and, therefore, activated to a variety of intermediates from soil or aquatic bodies to the air [11, 17].

Although there was limited use of synthetic organotellurium (OT) compounds in the past, they have turned a promising alternative for various applications, as evidenced by the increase in reports on OT compounds in the literature [18, 19]. Synthetic OT compounds have boomed in the last years, and their antioxidant, anti-inflammatory, antiproliferative, and immunomodulatory activities have been reported [18–20].

**Figure 1:**
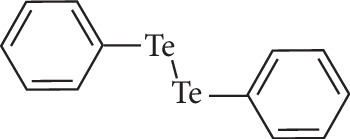
Chemical structure of diphenyl ditelluride.

In the present review, we emphasize the biological activities of an OT compound, diphenyl ditelluride (DPDT) (Figure 1), aiming to argue and discuss its contrasting antioxidant [21], cytotoxic [22], and antiproliferative [20, 23] effects.

## 2. Antioxidant and Chemopreventive Effects

The antioxidant effects of certain molecules are based on their ability to retard or inhibit oxidative damage. Their antioxidant role includes blocking oxidative reactions induced by highly reactive oxidant molecules—the so-called free radicals or reactive oxygen species (ROS)—that damage other molecules. The antioxidant properties of substances such as OT compounds can protect the biomolecules and cell components against oxidative damage [24–26]. OT compounds can act as ROS scavengers thus preventing the oxidation induced by highly reactive agents, including hydrogen peroxide and peroxyl radicals [19].

The oxidative stress plays an important role in the etiology of several conditions such as diabetes, autoimmune disorders, cardiovascular diseases, neurodegenerative diseases, and cancer [27]. The mammalian models have been extensively used for the evaluation of ROS-generated cellular damage and the protective effect of antioxidants [28]. In this context, the antioxidant properties of OT compounds and their potential use for treatment of oxidative stress-related conditions have been of interest to several research groups [19, 29, 30]. The efficacy of the organochalcogens in attenuating the oxidative stress in both *in vitro* studies and rodent models could be attributed to their ROS scavenging and glutathione peroxidase mimetic properties [25, 31, 32].

**Scheme 1:**
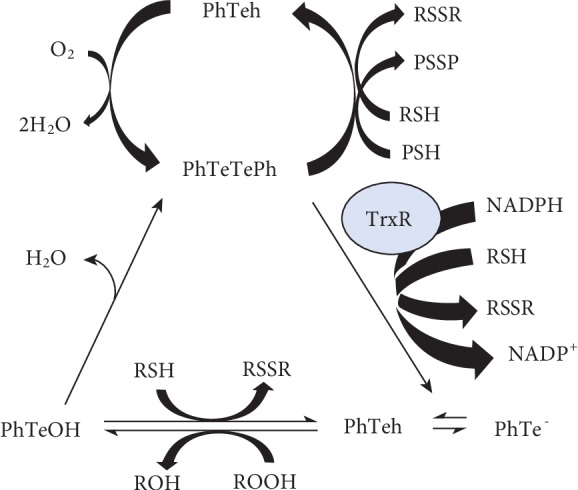
The proposed mechanism of the thiol peroxidation and thiol oxidation cycle of diorganotellurides. In the prooxidant pathway, the formation of the tellurol is associated with oxidation of low-molecular-mass (RSH) or protein-thiol (PSH) groups causing depletion of glutathione (GSH) through conjugation, oxidation, or export and/or protein loss of function. In the antioxidant pathway, organotellurium compounds decompose peroxides either as a substrate for mammalian thioredoxin reductase (TrxR) or as glutathione peroxidase-like activity via the formation of the tellurol/tellurate (PhTeh/PhTe^−^) (the scheme is reproduced from Puntel et al. (2012), under the Creative Commons Attribution License/public domain).

Puntel et al. (2012) intended that Te compounds have to be metabolized to tellurol/tellurate intermediates by different types of thiols, producing disulfides and regenerating the initial diorganotelluride as shown in Scheme 1, and proposed the mechanism of the thiol-peroxidase activity or thioredoxin-thiol-peroxidase-like activity of organotellurium compounds.

*In vitro* studies comparing the antioxidant properties of organochalcogenide compounds have demonstrated that their protective effects against lipid peroxidation reactions are mediated by free radical-scavenging activities (Table 1) [24, 26, 33]. In fact, 1.63 *μ*M DPDT inhibited lipid peroxidation in rat brain homogenates with higher efficacy than selenides and with similar efficacy to ebselen [34]. Also, DPDT provided protection against neurotoxicity and oxidative stress induction by 4-aminopyridine in mice [35]. The Na^+^/K^+^-ATPase activity in the rat brain significantly increased after treatment with low doses of DPDT, suggesting an antioxidant activity [36]. Further reports on the antioxidant activity of DPDT are summarized in Table 1.

The above results show that pretreatment with noncytotoxic concentrations of DPDT increased the survival of V79 cells exposed to methyl methanesulfonate, hydrogen peroxide, *t*-butyl hydroperoxide, and ultraviolet C radiation [21]. Furthermore, the pretreatment with the DPDT reduced oxidative DNA damage (oxidized pyrimidines and oxidized purines) detected by formamidopyrimidine DNA-glycosylase (Fpg) and endonuclease III (Endo III). Therefore, the protective effect of low-concentration DPDT preexposure can be explained by its antioxidant capacity in V79 cells (Table 1) [21].

**Figure 2:**
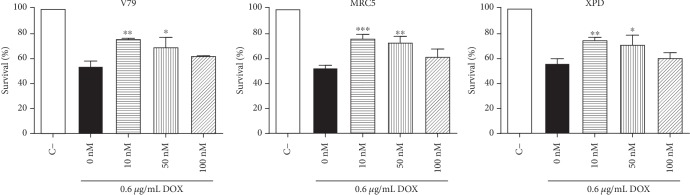
Protective effect of 2 h DPDT pretreatment in serum-free medium on doxorubicin-induced cytotoxicity in Chinese hamster fibroblasts (V79) as well as in human fibroblasts proficient (MRC5) and deficient (XPD) in NER evaluated by MTT assay 72 h after pretreatment. Data are reported as means ± SD of three independent experiments. Significantly different at ^∗^*p* < 0.05 and ^∗∗^*p* < 0.01 compared with cells treated with doxorubicin only (one-way ANOVA followed by Tukey test).

Some chemotherapeutic regimens have proposed the utilization of antioxidants to limit cytotoxicity and genotoxicity of free radical-inducing antitumor agents in normal tissues. The anthracycline doxorubicin (DOX) is a chemotherapeutic agent applied in the treatment of breast cancer and hematologic malignancies [37]. However, its use is limited due to induced cardiotoxicity via generation of ROS [37]. In view of the antioxidant effect of DPDT, we assessed the impact of low DPDT concentrations on DOX-induced cytotoxicity and genotoxicity in different cell lines (V79, MRC5, and XPD). For this purpose, the cell lines MRC5 and V79 were treated with DOX in the presence or absence of DPDT pretreatment and cell viability was evaluated using MTT assay. The pretreatment with 10 and 50 nM DPDT in V79, MRC5, and XPD cell lines increased the cell survival after 0.6 *μ*g/mL DOX treatment (Figure 2).

**Figure 3:**
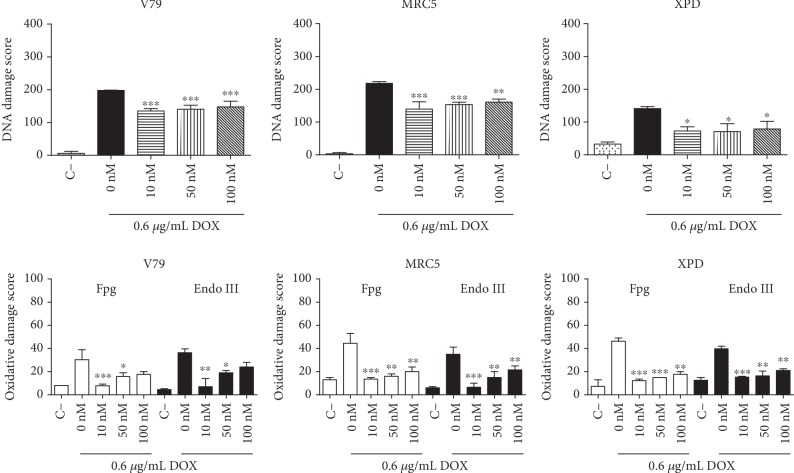
Antigenotoxic effects of 2 h DPDT pretreatment in serum-free medium on the genotoxicity of doxorubicin in Chinese hamster fibroblasts (V79) and human fibroblasts proficient (MRC5) and deficient (XPD) in NER, evaluated by comet assay and modified comet assay. Data are reported as means ± SD of three independent experiments. Significantly different at ^∗^*p* < 0.05, ^∗∗^*p* < 0.01, and ^∗∗∗^*p* < 0.001 compared with cells treated with doxorubicin only (one-way ANOVA followed by Tukey test). The damage index is an arbitrary score calculated from cells in different damage classes, which are classified by visual evaluation of the DNA migration length and the amount of DNA in the comet tail. The oxidative damage score represents the difference in the damage scores between cells incubated with the Fpg and Endo III enzymes and the cells incubated with the incubation buffer only.

**Figure 4:**
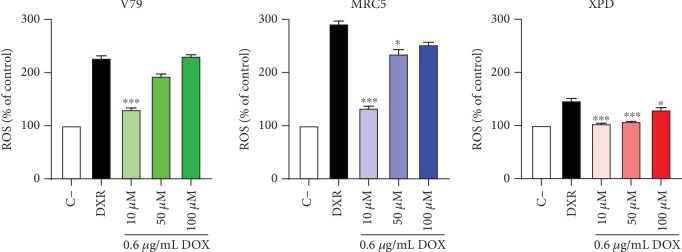
Effect of DPDT on DOX-induced ROS generation and ROS induction was evaluated via flow cytometry using DCFH-DA in cells pretreated with DPDT for 2 h in serum-free medium, followed by treatment with doxorubicin for 3 h. V79: Chinese hamster fibroblasts; MRC5 and XPD: human fibroblasts proficient and deficient in NER, respectively. Data are reported as means ± SD of three independent experiments. Significantly different at ^∗^*p* < 0.05, ^∗∗^*p* < 0.01, and ^∗∗∗^*p* < 0.001 compared with cells treated with doxorubicin only (one-way ANOVA followed by Tukey test).

The genotoxic effects induced by DOX were assessed by alkaline comet assay and enzyme-modified alkaline comet assay, which includes incubation with the enzymes Fpg and Endo III. DOX (0.6 *μ*g/mL) induced increase in the Fpg- and Endo III-sensitive sites (Figure 3), and after 3 h of DOX treatment, elevated intracellular ROS levels were detected via flow cytometry using dichlorofluorescein diacetate (DCFH-DA) (Figure 4). The effects of preexposure to low DPDT concentrations (10, 50, and 100 nM) on DOX-induced cytotoxicity and genotoxicity were evaluated, and all DPDT concentrations tested decreased DOX-induced genotoxicity (Figure 3) and ROS formation (Figure 4) in mammalian cells. These results demonstrate that low DPDT concentrations have a chemoprotective effect on DOX-induced DNA damage and do not affect its cytotoxicity in mammalian cells. This finding suggests possible utility of DPDT to prevent DOX-induced toxicity in normal tissues.

Compounds modulating cellular antioxidant defenses may influence the effectiveness of chemotherapy. Recently, some mechanisms related to DPDT antioxidant properties have been proposed in order to explain the chemoprotective effects [21]. DPDT can suffer nucleophilic attack in the Te atom thus interacting with thiol group-containing proteins and GSH [38, 39]. In agreement, DPDT was reported to inhibit the enzyme *δ*-aminolevulinic acid dehydratase in mice [38] and to decrease the GSH/GSSG ratio in yeast (50 *μ*M) and V79 cells (0.5 *μ*M) [22]. In contrast, DPDT induced an adaptive response increasing the sulfhydryl group content in the mouse brain [38]. As shown in Scheme 1, DPDT can cause depletion of GSH through oxidation, increasing the ROS formation or as a possible substrate for GSH conjugation. Reinforcing this hypothesis, it was shown that an organoselenium compound (structural analog of DPDT) is detoxified by conjugation with GSH in the rat liver [40, 41]. Thus, DPDT could modulate important endogenous antioxidant systems inducing GSH synthesis (Scheme 1) [22]. In part, this mechanism could explain previous results of our group showing DPDT antimutagenic and antigenotoxic effects [21], similar to that of DPDS (1.62–12.5 *μ*M) [41] but at lower concentrations.

For those compounds that display antioxidant activity, evaluation of their antimutagenic mechanisms of action is vital. The search for synthetic antimutagens is an important trend in the area of antimutagenicity research [42, 43]. Such compounds should act removing ROS through multiple antioxidant mechanisms, including modulation of the GSH level and activity of antioxidant enzymes such as superoxide dismutase (SOD) and catalase (CAT). Accordingly, DPDT significantly decreased the mutagenicity induced by two mutagens, MMS and UVC, possibly by restoring the GSH content, thus revealing its antioxidant and protector effects [21]. It was found that the antimutagenic potential of a variety of compounds could be attributed to their antioxidant activity (Table 1), and based on current knowledge, antioxidant activity is a desirable property since it can provide antimutagenic effects [21, 43].

## 3. Diphenyl Ditelluride Mechanisms of Antiproliferative Action in Cancer and Noncancer Cells

Cell death induction mechanisms are diverse, and it is broadly recognized that the effectiveness of Te compounds as anticancer agents is dependent on their chemical form and dose as well as on their redox state and the experimental model used [23, 33, 44, 45]. There is emerging evidence that cell death induced by Te compounds is associated with ROS formation, cell cycle arrest, induction of programmed cell death, and immunomodulatory effects [33]. Moreover, Te compounds may induce cell death by distinct pathways, either caspase-dependent or caspase-independent, depending on the chemical form and system studied [22, 23, 44]. Some mechanisms and actions of DPDT and other Te compounds are discussed below.

### 3.1. Stress Response and Cellular Targets

Due to increasing applicability of oxidative agents in the treatment of cancer, the use of antioxidant compounds for development of new anticancer agents has been a promising therapeutic strategy [44, 46]. ROS are essential for various biological processes in normal cells and can act in multiple signaling cascades in the cancer cell, regulating survival, proliferation, angiogenesis, and metastasis. Noncancer cells are characterized by a low basal level of ROS compared with cancerous cells [9, 42, 47]. In addition, the cancer cells develop an increased antioxidant capacity as a compensatory mechanism to escape the ROS-induced cell death, thus increasing their vulnerability to redox state-modulating agents [27]. The balance between oxidants and antioxidants determines the redox state of cells and tissues [9, 47]. Humans have developed highly complex antioxidant systems (enzymatic and nonenzymatic), such as GSH, thioredoxin (Trx) system, SOD, CAT, and peroxidase. These systems are dependent on either thiol antioxidants (GSH systems or the Trx system) [38].

**Scheme 2:**
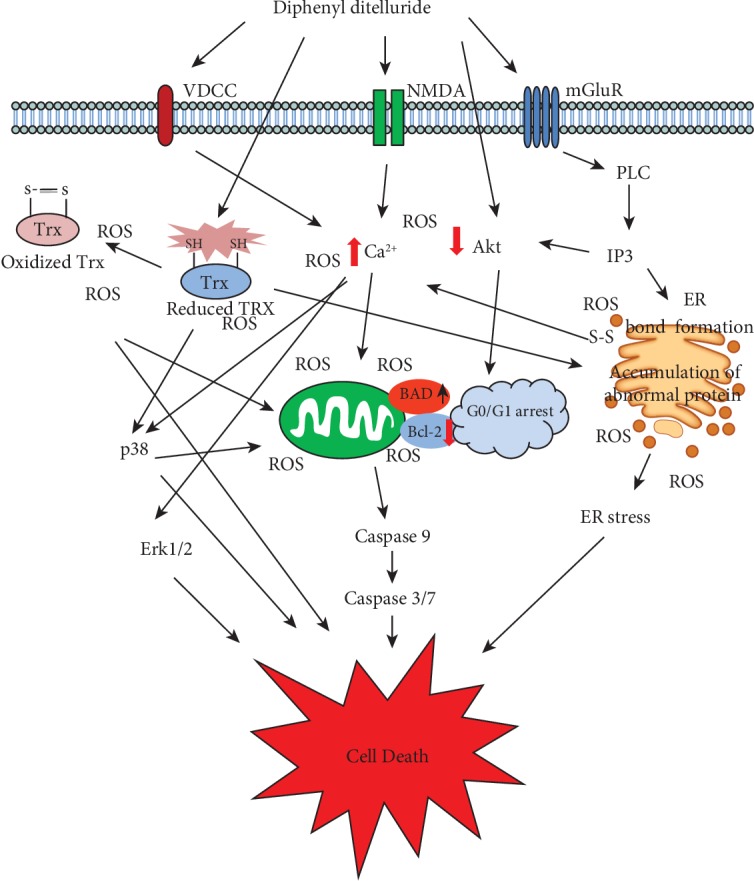
Mechanisms of diphenyl ditelluride antiproliferative action in cancer and noncancer cells. The high intracellular Ca^2+^ levels are correlated with an increase in Erk1/2 and p38MAPK phosphorylation. As a consequence, cellular redox imbalance and cell death were induced. DPDT, as a modulating agent of the cellular redox state, can interfere with the activity of different redox-active proteins (such as TRX), leading to redox imbalance and increasing their vulnerability to additional ROS-induced DNA damage and cell death. Also, DPDT can induce apoptosis and/necrosis by inducing as well as caspase activation. On the other hand, DPDT can induce cell death by decreasing Akt phosphorylation and activation of p38 and Erk1/2.

The GSH can act as a cofactor for several detoxifying enzymes; participate in amino acid transport across the plasma membrane; scavenge hydroxyl radical and singlet oxygen directly; regulate and activate transcription factors, such as AP-1and NF-*κ*B; and interact with other antioxidants regenerating (antioxidant network) their original properties, such as vitamins C and E [46]. The Trx system is a major antioxidant system integral to maintain the intracellular redox state and consists of Trx and TrxR, and the functions of this system in thiol-disulfide exchange reactions are essential to intracellular redox environment control, cellular growth, scavenging ROS, and apoptosis, thus displaying multiple roles in mammalian cells, including implications in cancer [48]. High concentration of Trx on plasma is raised in diseases associated with oxidative stress such as neurological disorders, arthritis, diabetes, and ischemia reperfusion injury and has been observed from many normal or tumoral cells [48, 49]. TrxR inhibition promotes a switch from an antioxidant to a prooxidant state and cell death induction; thus, TrxR inhibitors can be used for treatment of chemotherapy-resistant tumors (Scheme 1) [48–50]. TrxR-targeting may contribute to preventing resistance mechanisms, and there is evidence that the expression of TrxR correlates with apoptotic resistance in various cancer cell types [48]. In this manner, inhibition of TrxR and its related redox responses can contribute to adjuvant cancer treatment [50]. The Te compounds received special attention between other cancer cell redox modulators in relation to their promising chemotherapeutic potential [51]. Additionally, the chemotherapeutic potential of a number of effective synthetic and natural TrxR inhibitors are evaluated regarding induction of oxidative stress and apoptosis [48, 52]. Cyclodextrin-derived diorganyl tellurides were identified as novel inhibitors of TrxR with tumor growth inhibition capacities in submicromolar concentrations [52]. In addition, acute exposure of mice to 10 and 50 *μ*mol/kg DPDT caused TrxR inhibition (Table 2) (Scheme 2) [38].

### 3.2. Cytotoxic and Antiproliferative Effects

Different concentration thresholds for DPDT cytotoxicity were revealed for each biological model, *Salmonella typhimurium* (20 *μ*M), *Saccharomyces cerevisiae* (100 *μ*M), and V79 cells (1 *μ*M) [22, 23] (Table 2).

In another study, a significant decrease in cell viability was observed in a human colorectal adenocarcinoma cell line (HT-29) and heterogeneous human epithelial colorectal adenocarcinoma cells (Caco-2) treated at the concentration range of 62.5–1000 *μ*M DPDT and evaluated using MTT and luminescence assays (Table 2) [53]. The cytotoxic effects of 72 h DPDT treatment were studied also in acute promyelocytic leukemia (HL-60), human ileocecal adenocarcinoma (HCT-8), human glioblastoma (SF-295), and melanoma (MDAMB-435) cell lines.  Table 3 shows that the IC_50_ of DPDT was quite low for HL-60 (IC_50_: 0.03 *μ*g/mL), HCT-8 (IC_50_: 0.25 *μ*g/mL), and SF-295 (IC_50_: 0.28 *μ*g/mL) cell lines. The IC_50_ in the MDAMB-435 cancer cell line (2.16 *μ*g/mL) was higher than that in normal human peripheral blood mononuclear cells (CMSPH) (0.4 *μ*g/mL). DPDT was toxic in HL-60 cells (IC_50_: 0.03 *μ*g/mL) at a concentration close to that of the known chemotherapeutic agent DOX (IC_50_: 0.02 *μ*g/mL) and in an order of magnitude lower than the toxic DPDT concentration in normal CMSPH cells (0.4 *μ*g/mL). The cytotoxicity of DPDT is not due to unspecific damage to cell membranes since the hemolytic potential in erythrocytes was observed at a much higher concentration (244.25 *μ*g/mL) (Table 3).

**Figure 5:**
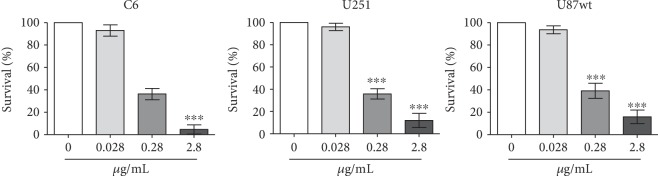
The clonogenic capacity of human glioblastoma (U87 and U251) and rat glial tumor C6 cell lines treated for 72 h with DPDT. Data are reported as means ± SD of three independent experiments. Significantly different at ^∗^*p* < 0.05, ^∗∗^*p* < 0.01, and ^∗∗∗^*p* < 0.001 compared with untreated control cells (one-way ANOVA followed by Tukey test).

The antiproliferative effects of DPDT in human glioblastoma U87 and U251 cell lines and in a rat glial tumor cell line (C6) was evaluated by clonogenic assay. The cells were treated for 72 h at 0.028, 0.28, and 2.8 *μ*g/mL DPDT for all cell lines. A DPDT concentration of 0.28 *μ*g/mL induced a greater reduction in cell viability, about 40–50%, for all cell lines (Figure 5). These results expand the possible utility of DPDT as an antiproliferative agent.

### 3.3. Effect on Cell Cycle and Cell Death

The administration of traditional chemotherapeutic agents inhibits the cell division inducing not only cell cycle alterations and apoptosis but other forms of nonapoptotic cell death such as necrosis, autophagy, and mitotic catastrophe. Since the most effective cancer treatment method used after surgery is chemotherapy, the search for new drugs with antiproliferative properties is currently ongoing [54, 55].

**Figure 6:**
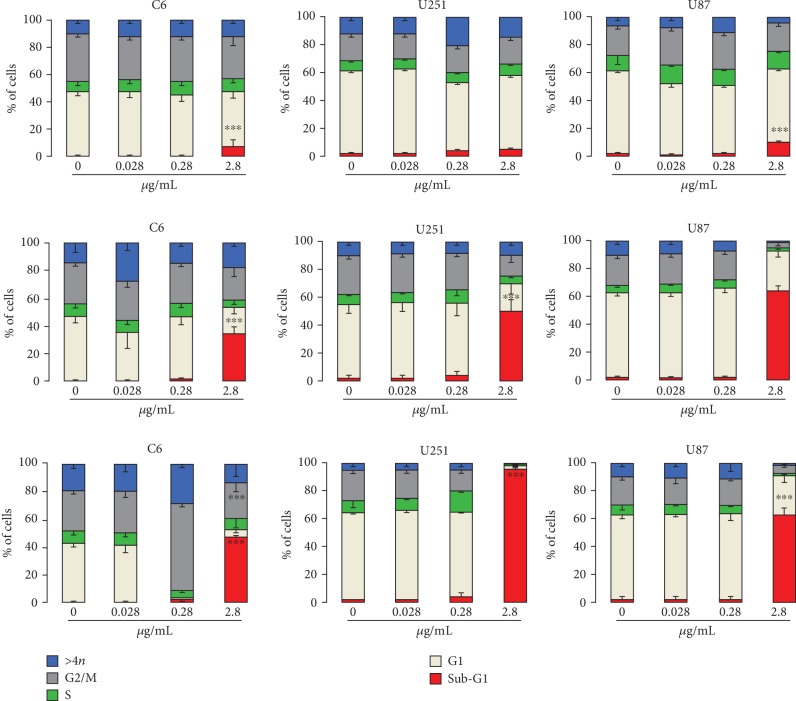
Effect of the DPDT treatment on the cell cycle distribution in U87, U251, and C6 cell lines. The cells were treated for 24, 48, and 72 h (first, second, and third lines, respectively) with DPDT at concentrations of 0.028, 0.28, and 2.8 *μ*g/mL. The values the % of sub-G1, G1, S, and G2/M phases and >4*n* cells, expressed as the means of four independent experiments, each performed in triplicate. The error bars indicate the standard error of the means. Significantly different at ^∗^*p* < 0.05, ^∗∗^*p* < 0.01, and ^∗∗∗^*p* < 0.001 compared with untreated control cells (one-way ANOVA followed by Tukey test).

In a human promyelocytic (HL-60) cell line, the DPDT treatment showed cell cycle alteration, an accumulation of S-phase cells after exposure to 1 *μ*M DPDT. This was the first study showing the effects of DPDT on the cell cycle (Table 2) [20]. In another study, we showed that treatment with 5 *μ*M DPDT resulted in the accumulation of S-phaseV79 cells [23]. Moreover, for all exposure times, 1 *μ*M DPDT did not affect the percentage of cells in any phase of the cell cycle (Table 2). We also evaluated the effects of DPDT (0.028–2.8 *μ*g/mL) on the progression of the cell cycle of different cancer cell lines (C6, U251, and U87) via flow cytometry analysis. After 24 h of treatment with DPDT (2.8 *μ*g/mL), the sub-G1 fraction of cells increased in the C6 and U87 cell lines (Figure 6). Furthermore, after 48 h DPDT (2.8 *μ*g/mL) treatment, an increase in sub-G1 cells was detected in all cell lines tested. It is important to note that 72 h of DPDT (0.28 *μ*g/mL) treatment also induced cell cycle arrest in the G2/M phase in C6 cells (Figure 6).

Similarly, in another study was reported an increase in the activity of caspases 3, 7, and 9 in HT-29 cells and in human colon fibroblasts (CCD-18Co) after treatment with DPDT (500–1000 *μ*M) (Table 2) (Scheme 2) [53]. In another study, Jorge et al. showed apoptosis and/or necrosis induction and an increase in the activity of caspases 3 and 7 at all treatment concentrations (1–10 *μ*M) in V79 cells [23]. On the other hand, DPDT treatment induces not apoptotic cell death in rat hippocampal astrocytes [56]. These differences may be due to variations in the GSH content of the different cell types (Table 2) [22]. Taking into account the ability of Te to bind thiol group-containing proteins, a decrease in the GSH/GSSH levels may be responsible for the induction of cell death (Scheme 2) [22, 23]. In this sense, DPDT was shown to reduce the activity of mitochondrial respiratory chain complexes (complexes I and II) by interaction with thiol groups. In this manner, DPDT can be considered as a putative apoptotic cell death inductor, acting via suppression of the pentose phosphate pathway caused by NADPH and thiol oxidation [57]. These results suggest that mitochondrial dysfunction could be an important factor in oxidative stress-related diseases (Scheme 2).

The results reported in DPDT studies lead us to infer the possible mechanisms of action of this drug and suggest its application as an antiproliferative agent in cancer therapy. As previously mentioned, results from our group demonstrated that DPDT can induce frameshift mutations in bacterial DNA and induce the formation of micronuclei in V79 cells [21, 22]. On the other hand, chemical substances with planar topologies are often capable of intercalation between the base pairs of DNA [58]. DNA-intercalating drugs can induce frameshift mutations in *Salmonella typhimurium* and *S. cerevisiae* and can be clastogenic in V79 cells [42]. In this way, the frameshift mutation induction by DPDT in bacteria and yeast and double-strand break induction in mammalian cells could be a result of intercalation activity as well as interaction with DNA topoisomerase enzymes (Table 2). In this sense, using *S. cerevisiae* mutants defective in topoisomerase enzymes, the results of Jorge et al. (2015) showed pronounced tolerance in the *top1Δ* strain to DPDT exposure. The same study also reported DPDT-induced inhibition of human TopoI activity *in vitro* using DNA relaxation assays [23]. These results suggest that DPDT could interact with the Top1p enzyme, inducing DNA lesions responsible for induced cell death.

Regarding the possibility of TopoI inhibition, the search for a noncamptothecin (CPT) TopoI inhibitor has been the target of several studies because of the limitation of CPT and its derivatives [59–62]. The aforementioned effects of DPDT, including cytotoxic effects and cell cycle arrest in the S- and G2/M phases, are consistent with those of other TopoI inhibitors (Scheme 2) [63]. The organotellurate immunomodulator AS101 induces G2/M arrest in myeloma cells and downregulates Cdc25C, Plk-1 (a serine/threonine kinase), and Ilk-1 (essential for regulating the activity of Akt) in mouse 5T33 myeloma cells [64, 65]. Halpert et al. demonstrated that AS101 targets several proteins and pathways in mice, such as pAkt, Bax, and Bcl-2 [66].

The intertwining of DNA strands and helices, produced during the essential cellular processes of replication, transcription, recombination, and chromosome segregation, must be resolved in order to maintain genome stability and cell viability. DNA topoisomerases supply an important solution for resolving such topological DNA problems [67]. However, they act through the formation of a covalent enzyme-DNA reaction intermediate, which is a potentially toxic lesion itself when stabilized. Indeed, targeting topoisomerase-DNA complexes has been widely explored in the identification and development of antibacterial and anticancer agents [67, 68]. These agents are known as “poison” inhibitors to indicate a mechanism of trapping topoisomerase and consequently forming a covalent enzyme-DNA complex, rather than a classic enzymatic inhibition mechanism, which would signify the lack of DNA binding or cleavage activity by the enzyme [69]. In this manner, increased production of drug-stabilized topoisomerase-DNA adducts increases the cytotoxic activity of a “poison,” while elevated levels of the enzyme could confer resistance to standard inhibitors. Furthermore, several DNA topoisomerases targeting drugs act as interfacial inhibitors (poisons) [67, 70], which are different from competitive (orthosteric) inhibitors and noncompetitive (allosteric) inhibitors because they interact at the interface between two or more molecules. The collision of DNA replication forks with the ternary complexes produce DNA double-strand breaks and cell death induction [70]. Our understanding of cell cycle arrest and cell death induction by DPDT and other OT compounds requires further investigation, but results obtained thus far lead us to some interesting insights. DPDT behaves like a molecule that presents several targets and could be of interest for cancer therapy research.

## 4. Systems Biology and Signaling

**Figure 7:**
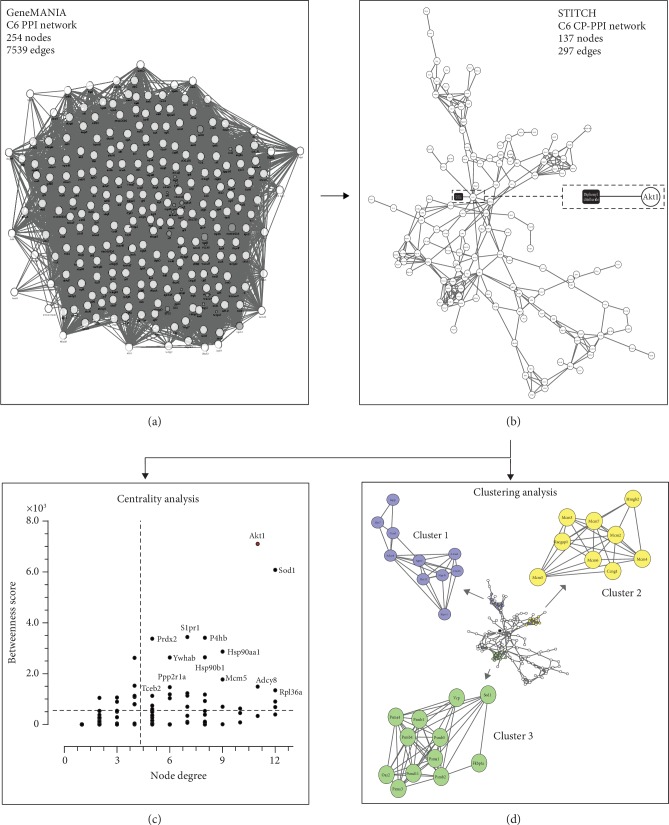
(a) C6 PPI network obtained from GeneMANIA software and cell line microarray data analysis. (b) C6 CP-PPI network showing interactions between DPDT and specific targets. (c) Network centrality analysis to define H-B nodes from the CP-PPI network. DPDT's H-B target appears in red. (d) Clustering analysis from the C6 CP-PPI network using ClusterONE [84, 85].

As described in previous sections, DPDT promotes different cellular phenomena. However, no molecular target associated with DPDT has yet been identified. We used a systems biology approach to predict putative cell signaling pathways or specific protein targets for DPDT. To this end, we prospected networks based on C6 cell line gene expression data. Contrast analysis was applied, and differentially expressed genes before simulation of DPDT treatment were selected using the rank product method, a technique based on calculating rank products (RP) from replicate experiments. We used three sample replicates of the C6 rat glioma cell line (untreated) from the GEO database (GSE1139 accession number) under culture conditions similar to those of our experimental model. For each sample, the average of the signal between the same probes was calculated and applied to the normalized microarray data using the limma package in the R/Bioconductor software [71, 72]. The parameters used to run the RP were the following: permutation = 1000 and *p* value ≤ 0.01. After this, we obtained initial C6 protein-protein interaction (PPI) networks based on physical interactions from the GeneMANIA prediction server [72]. The network is composed of 254 nodes and 7539 interactions (Figure 7(a)). To simulate DPDT action, we used STITCH software [73] which allows construction of chemical-protein (CP) and PPI networks (please see Figure 7(b)). Based on this strategy, a network designated C6 CP-PPI was obtained. The analysis showed DPDT interaction with a molecular target, Akt serine/threonine-protein kinase, which is involved in the regulation of multiple signaling pathways involved in cell metabolism and angiogenesis (Scheme 2) [74]. It is also a member of the most frequently activated cell proliferation, drug resistance, and survival pathways in several cancer types [75–77].

In addition, to define Akt interconnectivity and relevance in the C6 CP-PPI network, we analyzed some topological parameters. Firstly, we applied CentiScaPe software to explore node degree and betweenness centralities [78]. Based on this analysis, Akt appeared as a major hub-bottleneck node when compared with the other proteins in the network (Figure 7(c)). Similarly, clustering showed that Akt is a point with high information flux (Figure 7(d)).

Our strategy is valid to explore potential targets for DPDT. However, targets for this compound appear to be different according to the exposed cell type. Heimfarth et al. showed DPDT-induced hyperphosphorylation of glial fibrillary acidic protein, vimentin, and neurofilament subunits from glial cells [79]. The authors reported that excessive Ca^2+^ influx activated protein kinase A and protein kinase C in astrocytes, causing the hyperphosphorylation of glial fibrillary acidic protein and vimentin. These disrupt the organization of actin stress fibres formed by endogenous RhoA activation and led to altered cell morphology. In neurons, the overexpression Ca^2+^ levels activated Erk and p38MAPK, beyond the protein kinase A and protein kinase C, provoking hyperphosphorylation of neurofilament subunits (Table 2), and as a consequence must have caused cellular redox imbalance, increasing ROS and inducing cell death (Scheme 2).

Systems biology strategies combined with *in vitro* and *in vivo* studies could elucidate the molecular mechanisms responsible for the multiple effects of DPDT. To this end, future investigations are necessary to establish the suitability of DPDT application for targeted cancer therapies.

Many authors have shown that DPDT treatment induces different cell death pathways in several model systems, including apoptosis and/or necrosis, but the regulating mechanisms of this agent look very complex. Based on the results discussed above, we can summarize the mechanisms of antiproliferative action in cancer and noncancer cells of DPDT action in Scheme 2.

## 5. Conclusion

**Figure 8:**
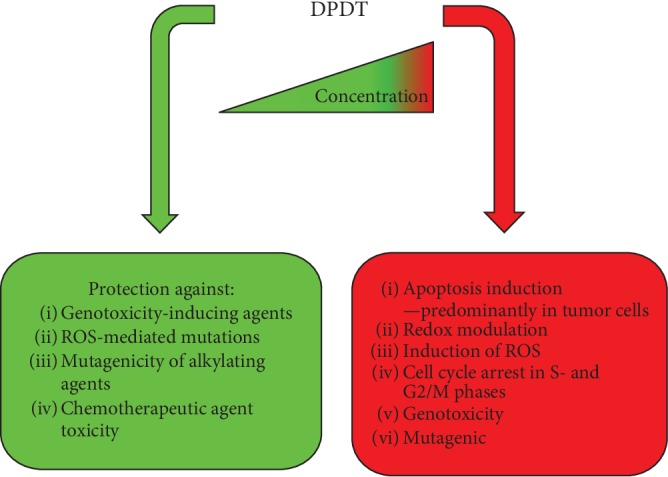
Biological effects of diphenyl ditelluride. Low concentrations of DPDT showed protective effects that could be attributed to its antioxidant capacity. DPDT at moderate concentrations showed selective cytotoxic effects, inducing preferential apoptosis in tumor cells. The observed cytotoxic effects can be explained by increased ROS formation and redox modulation. High concentrations of DPDT-induced toxicity and mutagenicity.

In the past decade, several organometallic compounds have entered clinical trial owing to their unique redox-modulating features and great potential in cancer therapy [80, 81]. The toxicology of DPDT has been evaluated by few laboratories, by either animal studies or assessment of cell growth inhibition *in vitro*. It is important to note that in HL-60 cells, the cytotoxic effect of DPDT (IC_50_: 0.03 *μ*g/mL) was observed at a similar concentration range to that of the antitumor agent DOX (IC_50_: 0.02 *μ*g/mL). This concentration is more than an order of magnitude lower compared to the toxic DPDT concentration in normal CMSPH cells (0.4 *μ*g/mL). Furthermore, the hemolytic potential in erythrocytes was observed at high concentration, and the cytotoxicity of DPDT cannot be attributed to unspecific damage to cell membranes. Albeit the conventional prejudice concerning organotellurium compounds, DPDT use should be considered with caution because of its high reactivity and toxicity at relatively low concentrations. The exact nature of DPDT cytotoxic effects remains unclear, although some mechanisms have been proposed that differ depending on the system studied. New trials regarding toxicity, checkpoint activation, and mechanisms of cell death induction by DPDT should be explored in a greater number of cell lines. Moreover, its topoisomerase inhibition potential should be further investigated, keeping in mind that this compound probably presents several distinct mechanisms of action. DPDT have particular chemistry with the thiol which is related to many of the biological effects observed so far. The DPDT depletes GSH because of oxidation and/or as a possible substrate for GSH conjugation and could modulate cellular antioxidant defenses inducing GSH synthesis. The dual action of DPDT (protective and toxic) opens possibilities for distinct applications in cancer treatment (Figure 8). In neurons, the high intracellular Ca^2+^ levels activated Erk and p38MAPK, beyond the PKA and PKC, provoking hyperphosphorylation of neurofilament subunits, and as a consequence must have caused cellular redox imbalance, increasing ROS and inducing cell death. ROS-generating drugs induce cancer-specific cytotoxicity by elevated endogenous ROS production in preclinical studies [82, 83]. Thus, low DPDT doses may be useful in the development of adjuvant therapies or rational combinations that may be predicted to have synergistic or additive effects in combination with currently used chemotherapeutics. In addition, the role of DPDT in the prevention of ROS-mediated diseases warrants additional studies to better understand and elucidate the mechanisms of its antioxidant and prooxidant activities before further developments of the uses of tellurium compounds in biology and medicine.

## Figures and Tables

**Table 1:** The chemopreventive effects of diphenyl ditelluride.

Model	DPDT (*μ*M)	Effects	Inducing agent	Reference
Rat brain	*1.63*	Inhibition of thiobarbituric acid reactive species (TBARS) formation by 50%	Quinolinic acid (QA) and sodium nitroprusside (SNP)	[24]
Rat brain	150 (*μ*mol/kg)	Neuroprotective activity	4-Aminopyridine	[35]
Rat brain	1–4	Increased Na^+^/K^+^-ATPase	—	[36]
V79 cell line	0.01–0.1	Reduced cytotoxicity; reduced DNA damage, micronucleus, and ROS formation	Hydrogen peroxide (H_2_O_2_), *t*-butyl hydroperoxide (*t*-BOOH), methyl methanesulfonate (MMS), and UVC	[21]
V79, MRC5, and XPD cell lines	0.01–0.1	Reduced DNA damage and ROS formation	Doxorubicin (DOX)	Figures 2–4

**Table 2:** Diphenyl ditelluride mechanisms of action in cancer and noncancer cells.

Model	DPDT	Results	Reference
V79 cell line	*0.5–1 μ*M	Reduced superoxide dismutase (SOD) activity; increased TBARS and ROS formation	[21]
V79 cell line	0.5*–*50 *μ*M	Increased TBARS; reduced GSH : GSSH ratio	[21]
Mouse brain	*10–50 μ*mol/kg	Reduced SOD, catalase (CAT), glutathione peroxidase (GPx), glutathione reductase, and thioredoxin reductase (TrxR) activities	[38]
V79 cell line	1–50 *μ*M	Cytotoxic and genotoxic effects	[22]
Caco-2 cell line	62.5–1000 *μ*M	Antiproliferative effect	[53]
HL-60 cell line	1 *μ*M	Antiproliferative effect, apoptosis induction, and accumulation of S-phase cells	[21]
HL-60, HCT-8, SF-295, MDAMB-43, and CMSPH cell lines	0.03–2.16 *μ*g/mL	Antiproliferative effect	Table 3
C6, U87, and U251 cell lines	0.28–2.8 mM	Antiproliferative effect; increase of G2/M phase cells in the C6 cell line and in sub-G1 phase cells in C6, U87, and U251 cell lines	Figures 5 and 6
HT-29 and CCD-18Co cell lines	500–1000 *μ*M	Apoptosis induction; increase in caspases 3/7 and caspase 9 activity	[53]
V79 cell line	1–10 *μ*M	Increased caspase 3/7 activity, apoptosis, necrosis, and inhibition of human TopoI activity	[23]
C6 cell line (systems biology)	—	Interaction with Akt1 protein kinase	Figure 7
Astrocytes and neurons	0.1–0.5 *μ*M	Hyperphosphorylation of GFAP and vimentin mediated by NMDA receptors and L-VDCCs; activation of Erk and p38MAPK	[79]

**Table 3:** Cytotoxic effects of diphenyl ditelluride compared with those of doxorubicin on different cell lines.

Compound (*μ*g/mL)	Cell line					
	HL-60	HCT-8	SF-295	MDAMB-435	CMSPH	Erythrocyte
DPDT (IC_50_)	0.03	0.25	0.28	2.16	0.40	>100 *μ*g/mL (244.25)
DOX (IC_50_)	0.02	0.01	0.24	0.48	0.97	ND^∗^

^∗^Not determined.
